# Paradoxical cardiotoxicity of intraperitoneally-injected epigallocatechin gallate preparation in diabetic mice

**DOI:** 10.1038/s41598-018-25901-y

**Published:** 2018-05-18

**Authors:** Nora O. Abdel Rasheed, Lamiaa A. Ahmed, Dalaal M. Abdallah, Bahia M. El-Sayeh

**Affiliations:** Department of Pharmacology and Toxicology, Faculty of Pharmacy, Cairo University, Cairo, Egypt

## Abstract

Numerous clinical and bioavailability studies addressed epigallocatechin gallate (EGCG) beneficial effects; however, our previous work revealed EGCG-induced nephrotoxicity in the presence of diabetes. In this study, the potential myocardial toxicity of EGCG preparation (100 mg/kg/day, IP; 4 days) in diabetic mice injected with streptozotocin (STZ; 150 mg/kg, IP) was investigated. Diabetic mice receiving EGCG preparation showed electrocardiographic changes in addition to elevation of both serum creatine kinase-MB and troponin-I levels accompanied by microscopic myocardial damage. Additionally, myocardial NADPH oxidase, lipid peroxides and nitrotyrosine were increased in the vicinity of decreases of nuclear factor erythroid 2-related factor 2, hemeoxygenase-1, reduced glutathione, total antioxidant capacity, glutathione peroxidase and reductase and heat shock protein 90. Moreover, in diabetic mice, EGCG preparation increased myocardial nuclear factor-kappa B and tumor necrosis factor-alpha in addition to pronounced overexpression of inducible nitric oxide synthase and active caspase-3. Therefore, this study substantiates that EGCG-mediated deterioration compromises diabetes-induced cardiotoxicity to solidify our previous report for its potential nephrotoxicity in the same experimental setting.

## Introduction

Diabetes mellitus presents from a constellation of heterogeneous malfunctions witnessed by episodes of hyperglycemia and glucose intolerance resulting from lack and/or defective insulin or insulin action. This type of metabolic syndrome arises from derangements in the regulatory systems for storage and mobilization of metabolic fuels^1^. Of note, diabetics have a 2 to 4 fold higher risk for cardiovascular events, where around 80% of diabetes-associated deaths are linked to cardiovascular disease (CVD)^2^. As such, diabetes is regarded as a coronary heart disease risk equivalent, i.e.; with a similar risk as an individual with a previous CVD event^3^. Hyperglycemia is known to aggravate intracellular reactive oxygen species (ROS) formation which induces inflammation and eventually apoptosis^4^. Most of the potential health benefits associated with green tea consumption have been linked to epigallocatechin-3-gallate (EGCG), a major catechin found in green tea^5^. EGCG exhibits anti-oxidant, anti-inflammatory and anti-apoptotic potentials^6–8^. Cardioprotective effects of EGCG were also reported in previous experimental studies of hypercholesterolemia and doxorubicin-induced injury^9,10^. On the other hand, several reports linked the consumption of EGCG containing supplements to acute hepatitis, and hence hepatotoxicity^11–13^. Drug-induced oxidative stress is implicated as a mechanism of toxicity in numerous tissues and organ systems, including liver, kidney, ear, and cardiovascular and nervous systems^14^. After being absorbed and passed onto the systemic circulation, EGCG undergoes oxidation in liver either enzymatically or non-ezymatically (e.g. oxidation from ROS leaked from the mitochondria) resulting in the formation of a reactive intermediate generating more ROS which react with cellular macromolecules resulting in hepatotoxicity^15^.

Experimentally, apart from its liver injurious effect when administered orally, high doses of EGCG were found to induce renal and intestinal damage when given in diet^15,16^. Furthermore, it was reported that natural products may act differently in health compromised affected subjects^17^. Noteworthy, in the presence of predisposing conditions, such as colitis, fever and diabetes, EGCG initiated paradoxical effects that were not obvious in healthy animals^16,18,19^. In fact, the administration of EGCG in the presence of lipopolysaccharide-induced fever or dyslipidemia in mice resulted in hepatotoxicity, while in colitic mice it induced nephrotoxicity^16,18^. These effects are linked to the pro-oxidant nature of EGCG which is attributed to its catechol structural moiety which has low half peak oxidation potential where high concentration of EGCG can undergo self-oxidization and function as prooxidant by producing superoxide anion, hydroxyl radicals, hydrogen peroxide, and quinonoid intermediates causing cytotoxicity^20,21^.

Based on the data of our previous work signifying nephrotoxic effect of EGCG preparation in mice mediated by oxidative stress, inflammation and apoptosis, the current study was undertaken to delineate the potential cardiotoxic effect of Teavigo^®^ consisting of 94% EGCG in diabetic mice.

## Materials and Methods

### Animals

Male Swiss albino mice (25–30 g; Faculty of Pharmacy, Cairo University, Cairo, Egypt) were housed under controlled temperature (25 ± 2 °C) and 12 h light/dark cycles with free access to a standard rodent chow diet and water *ad libitum*. The study was approved by the Ethics Committee for Animal Experimentation (Faculty of Pharmacy, Cairo University; PT 1271) and complies with the Guide for Care and Use of Laboratory Animals (USA NIH, 2011).

### Chemicals

The highly purified extract (94% EGCG) Teavigo^®^ (DSM Nutritional Products, Kaiseraugst, Switzerland) and streptozotocin (Sigma–Aldrich, MO, USA) were used in this study.

### Experimental design

Mice were randomly divided into 4 groups (n = 16–30  mice each), where saline was injected intraperitoneally in mice to serve as the normal control group. A single dose of STZ (150 mg/kg, IP) was injected in animals to serve as the diabetic control group^22^. In addition, EGCG preparation (100 mg/kg, IP; 4 days) was administered in normal or diabetic animals representing either EGCG or STZ + EGCG groups, respectively^19^. The used dose of EGCG preparation was selected based on a pilot study where different doses of EGCG preparation (50, 100, 150 and 200 mg/kg IP) were investigated to detect the highest safe dose in normal animals which caused no myocardial injury based on measurement of serum troponin-I level. Only EGCG150 showed significant high troponin-I level which indicates its cardiotoxic potential whereas EGCG 200 killed all injected animals which confirms its lethality. Accordingly, EGCG 100 was the highest dose which showed no myocardial damage and hence was selected to be used in the main study. Notably, EGCG preparation was injected 48 h after diabetes induction, where mice with blood glucose level ≥200 mg/dl (One Touch Select Glucometer, Life Scan, Pennsylvania,USA) were selected.

Twenty four hours after the last dose of EGCG administration, animals were weighed. Mice were then anesthetized with thiopental (60 mg/kg, i.p.) and kept warmed with a heating lamp to prevent the incidence of hypothermia. Subcutaneous peripheral limb electrodes were inserted for electrocardiographic recording (HPM 7100, Fukuda Denshi, Tokyo, Japan) to determine heart rate (HR) and corrected QT interval (QTc). At the end of experiment, blood was collected under anesthesia and sera were separated for the estimation of creatine kinase-MB (CK-MB)and troponin-I levels. Animals were then euthanized and hearts were rapidly excised, washed with ice-cold saline, dried and weighed. In the present study, 2 equal sets of experiments were conducted; one was used for weight assessment and ECG recording and the heart was homogenized in saline to prepare 5% homogenate for estimation of oxidative stress and inflammatory markers. The other set was used for blood sampling, western blot analysis and histopathological examination.

### Biochemical examination

#### Serum creatine kinase-MB

Serum CK-MB was assessed using a commercially available kit (Stanbio, USA). Results were estimated kinetically at 340 nm using spectrophotometer (Thermo electron corporation, England) and expressed as U/l.

#### Serum troponin-I

Serum troponin was estimated using ELISA kit (Kamiya Biomedical Company, Washington, USA). The procedure of the used kit was performed according to the manufacturer’s instructions and the results were expressed as ng/ml.

#### Oxidative stress and inflammatory markers

ELISA kits were used for the assessments of heart NADPH oxidase and nuclear factor erythroid 2-related factor 2 (Nrf2; Cusabio, Wuhan, China), hemeoxygenase-1(HO-1; Enzo Life Sciences, New York, USA), heat shock protein 90 (HSP 90) and nuclear factor-kappa B (NF-κB; Eiaab,Wuhan, China), tumor necrosis factor-alpha (TNF-α; Raybio, Georgia, USA) and nitrotyrosine (MyBioSource, San Diego, USA). The procedure of the used kits was performed according to the manufacturer’s instructions and the results were expressed as ng/mg protein for NADPH oxidase, Nrf2, HSP90 and TNF-α and as μg/mg protein for HO-1 and NF-κB. Results of nitrotyrosine was also expressed as pmol/mg protein.

Moreover, reduced glutathione (GSH), glutathione peroxidase (GPx), glutathione reductase (GR) and thiobarbituric acid reactive substances (TBARS) were assessed using Biodiagnostic kits (Egypt). Procedures were performed according to manufacturer’s instructions and results were expressed as mg/g wet tissue for GSH, nmol/mg protein for TBARS and U/mg protein for GPx and GR. Total antioxidant capacity (TAC) was measured colorimetrically using Zen-Bio kit (North Carolina, USA) and results were expressed as mmol/mg protein. The protein content in tissue homogenates was determined according to the method of Lowry *et al*.^23^.

#### Western blot analysis of inducible nitric oxide synthase

Part of heart was homogenized in lysis buffer and quantified for protein levels using a Bicinchoninic acid protein assay (BCA) kit (Thermo Fisher Scientific Inc., USA). Protein expression was assessed as previously described^24^ using anti-iNOS antibodies (Stressgen Biotechnologies, Victoria, British Columbia, Canada). The amount of protein was quantified by densitometric analysis of the autoradiograms using a scanning laser densitometer (Biomed Instrument Inc., USA). Results were expressed as arbitrary units after normalization for beta-actin (β-actin) protein expression.

### Histopathological examination

At the end of the experimental period, the apex of heart was isolated, rinsed in ice-cold saline and immediately fixed in 10% formalin. The specimens were processed for paraffin embedding, and 5μm sections were prepared and then stained with hematoxylin and eosin (H&E; x400).

### Active caspase-3 immunohistochemical examination

Immunohistochemical examination of active caspase-3 was carried out on 5 μm thick deparaffinized sections incubated in a 0.01 M sodium citrate solution (pH 6) at 120 °C for 10 min, followed by a 2 h cool-down. The sections were then treated with the proteolytic enzyme proteinase K (Dako, Copenhagen, Denmark), washed in phosphate buffered saline (PBS) for 5 min and incubated with a primary antibody against caspase-3 (Novus Biologicals, Colorado, USA) for 60 min at 37 °C. After washing in PBS, a secondary antibody (Novus Biologicals, Colorado, USA) was applied for 60 min, visualized with 3,3′-diaminobenzidine (DAB) chromagen (Dako, Copenhagen, Denmark) and counterstained with hematoxylin (x400).

### Statistical analysis

All data obtained were presented as mean ± SEM. Results were analyzed using one-way analysis of variance (ANOVA) followed by Tukey-Kramer multiple comparisons test. Statistical analysis was performed using GraphPad Prism^©^ software (version 6.01; Graph Pad Software, California, USA). For all the statistical tests, the level of significance was fixed at *P* < 0.05.

## Results

### EGCG induced changes in heart to body weight ratio as well as perturbations in electrocardiographic measurements, myocardial biomarkers and histological examinations in diabetic mice

**Figure 1:**
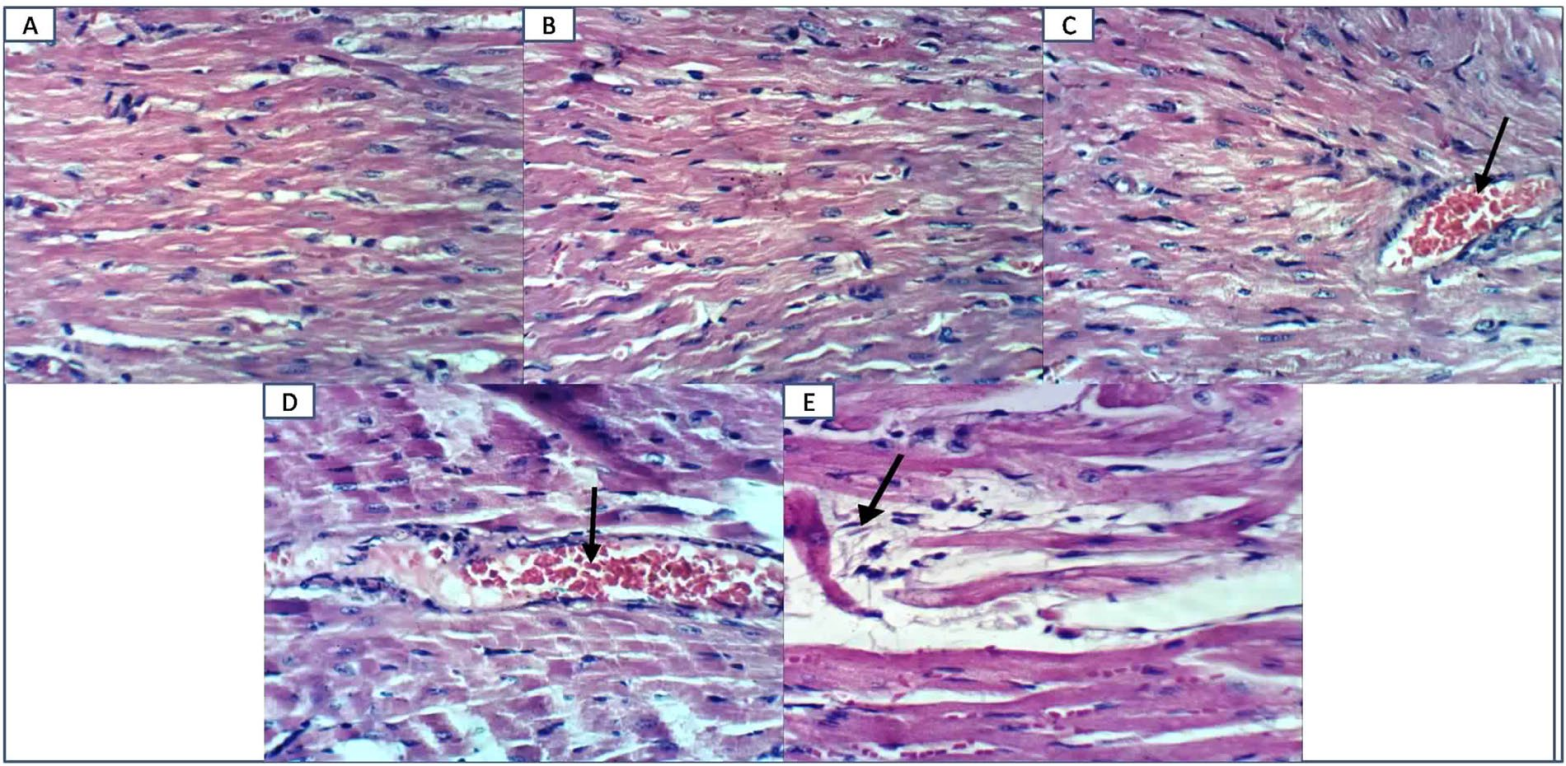
Effect of EGCG on STZ- induced changes in histological examination (H&E x400). (**A**) Heart of a normal mouse showing normal cardiomyocytes. (**B**) Heart of a mouse from EGCG group showing normal cardiomyocytes. (**C**) Heart of a diabetic mouse showing moderate congestion of myocardial blood vessels. (**D**) Heart of a diabetic mouse receiving EGCG showing severe congestion of myocardial blood vessels. (**E**) Heart of a diabetic mouse receiving EGCG showing moderate intermuscular edema.

Myocardial alterations were assessed by measurements of heart to body weight ratio as well as HR and QTc. Serum CK-MB and troponin-I levels were also estimated as markers of myocardial damage. EGCG group showed no alteration in these parameters compared to normal group. STZ increased heart to body weight ratio and QTc whereas it decreased HR. Moreover, this group showed elevation of serum CK-MB and troponin-I levels. On the other hand, administration of EGCG to diabetic mice markedly deteriorated these parameters compared to diabetic group confirming that EGCG preparation potentiated diabetes-induced myocardial damage (Table 1). Moreover, EGCG administration to normal mice showed no histopathological alteration compared to the normal control mice. However, diabetic mice showed moderate congestion of myocardial blood vessels whereas EGCG administration to diabetic mice caused severe congestion of myocardial blood vessels and moderate intermuscular edema verifying that EGCG preparation potentiated diabetes-induced myocardial damage (Fig. 1).

**Table 1:** Effect of EGCG on STZ-induced changes in body weight, heart to body weight ratio and electrocardiographic measurements as well as serum CK-MB and troponin- I levels.

Groups	Normal (saline)	EGCG	STZ	EGCG + STZ
Final body weight (g)	26.80 ± 0.58	26.00 ± 0.84	23.20^*@^ ± 0.58	23.20^*@^ ± 0.58
Heart/ body weight ratio (mg/g)	4.48 ± 0.13	4.76 ± 0.19	6.08^*@^ ± 0.20	6.72^*@^ ± 0.23
HR (bpm)	421.80 ± 8.83	412.00 ± 8.81	376.30^*@^ ± 9.54	340.30^*@#^ ± 8.77
QTc (ms)	35.17 ± 0.87	33.83 ± 1.11	41.67^*@^ ± 1.12	50.00^*@#^ ± 1.86
CK-MB (u/l)	66.40 ± 6.27	66.40 ± 5.64	107.60^*@^ ± 5.08	189.60^*@#^ ± 12.19
Troponin-I (ng/ml)	0.66 ± 0.03	0.58 ± 0.03	1.92^*@^ ± 0.06	2.99^*@#^ ± 0.04

HR, heart rate; bpm, beats per min; Qtc, corrected QT interval.

Each value represents the mean of 6–8 experiments ± S.E.M. *P < 0.05 vs. normal, ^@^*P* < 0.05 vs^.^ EGCG, ^#^*P* < 0.05 vs. STZ.

### EGCG exaggerated oxidative stress in diabetic mice

**Figure 2:**
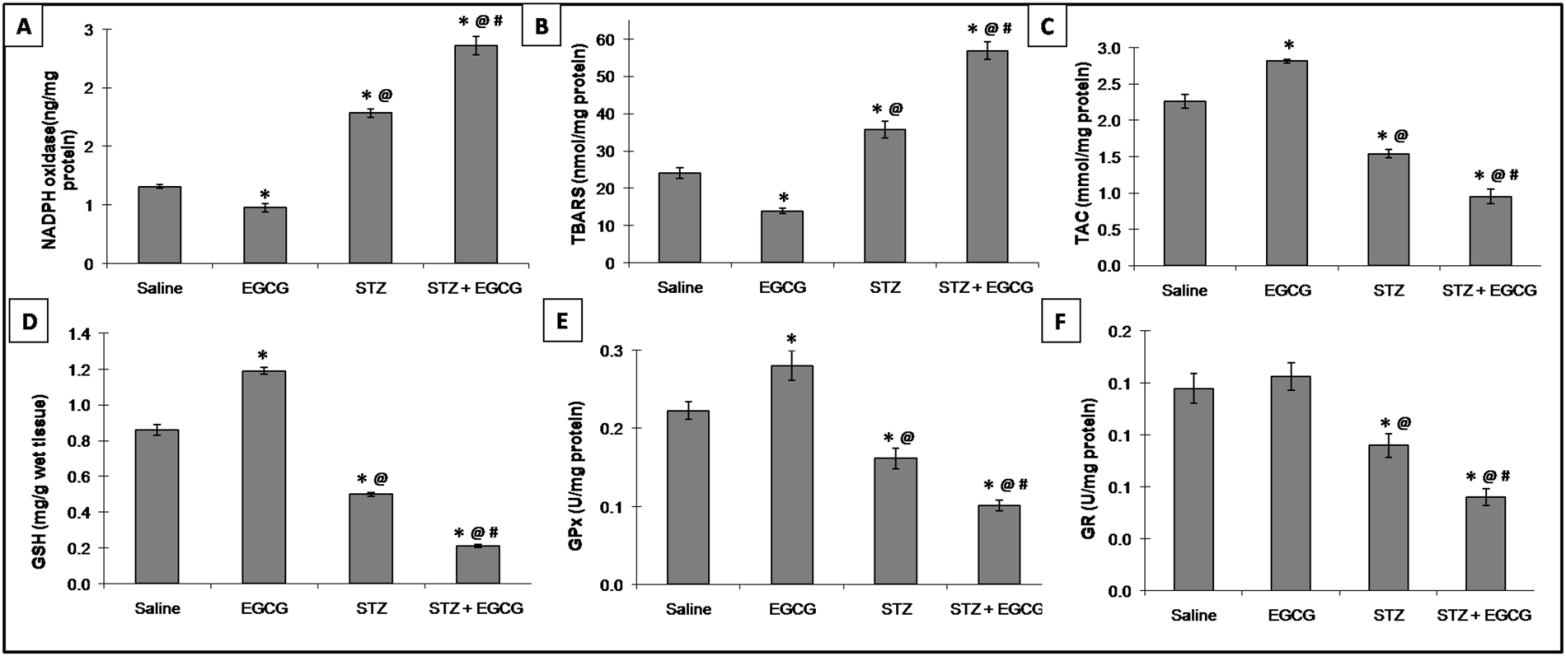
Effect of EGCG on STZ-induced changes in (**A**) NADPH oxidase, (**B**) TBARS, (**C**) TAC, (**D**) GSH, (**E**) GPx and (**F**) GR. Each value represents the mean of 6–8 experiments ± S.E.M. *P < 0.05 vs. normal, ^@^*P* < 0.05 vs. EGCG, ^#^*P* < 0.05 vs. STZ.

**Figure 3:**
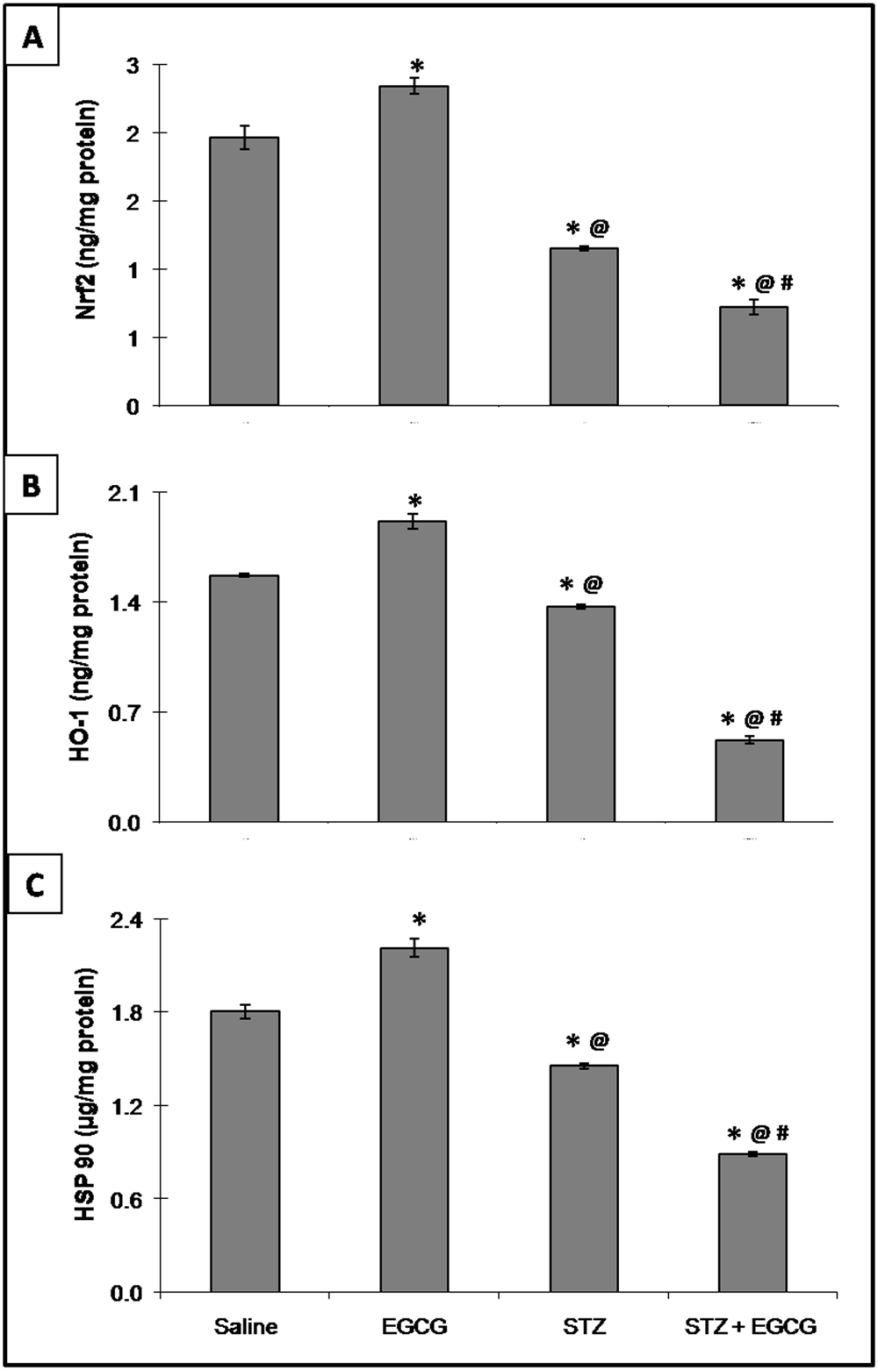
Effect of EGCG on STZ-induced changes in (**A**) Nrf2, (**B**) HO-1 and (**C**) HSP 90. Each value represents the mean of 6–8 experiments ± S.E.M. *P < 0.05 vs. normal, ^@^*P* < 0.05 vs. EGCG, ^#^*P* < 0.05 vs. STZ.

In normal mice, EGCG significantly decreased NADPH oxidase, TBARS and nitrotyrosine contents with increases in TAC, GSH, GPx, Nrf2, HO-1, and HSP 90 contents indicating restrained ROS generation. On the other hand, diabetic mice receiving EGCG have shown increased NADPH oxidase, TBARS and nitrotyrosine associated by decreases in TAC, GSH, GPx, GR, Nrf2, HO-1 and HSP 90 cardiac contents. These effects were more profound than those in STZ animals, signifying the worsening of the oxidative stress status with a shifted balance towards a pro-oxidative milieu by STZ and EGCG administration (Figs 2 and 3).

### EGCG augmented inflammation in diabetic mice

**Figure 4:**
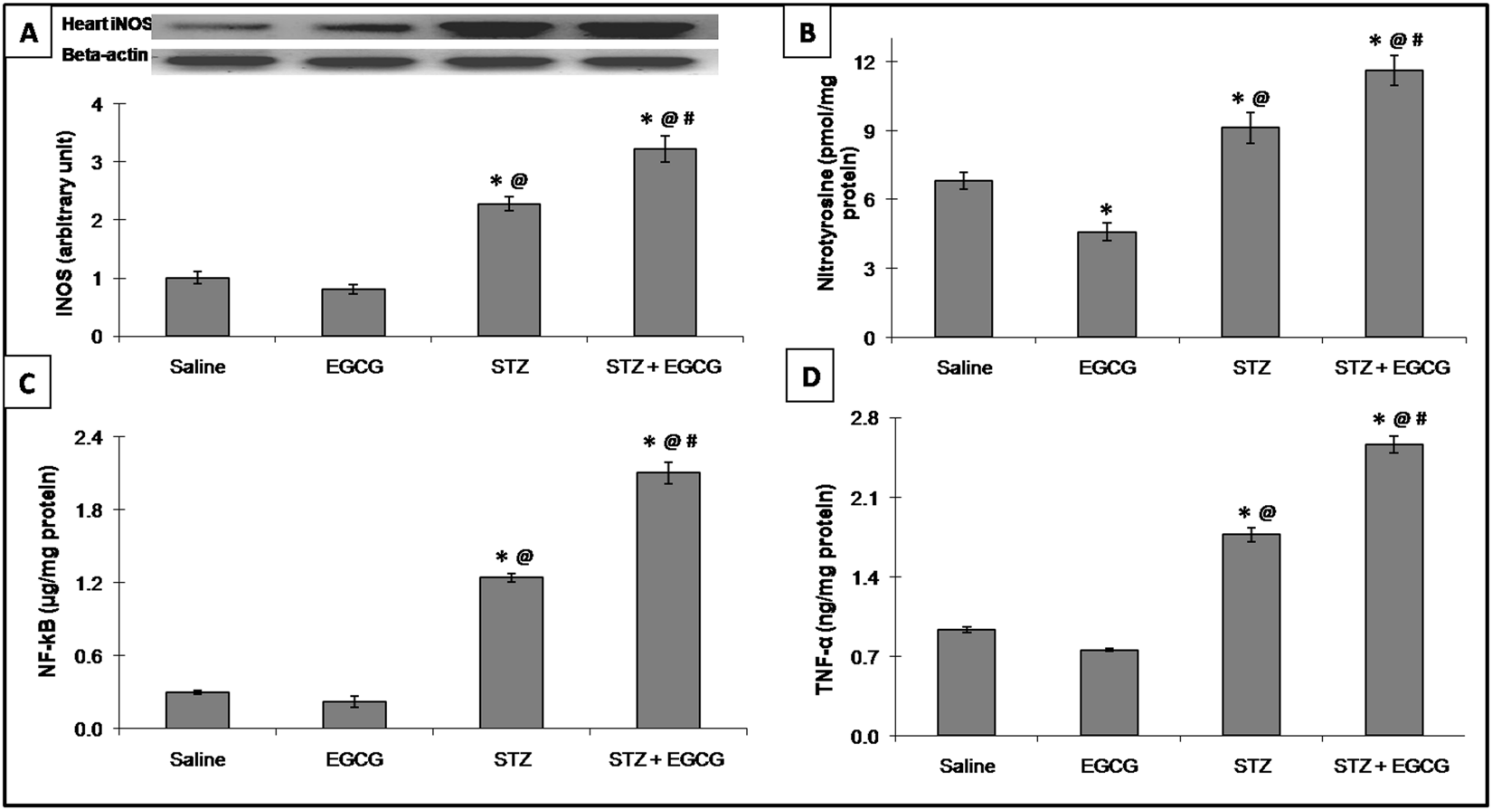
Effect of EGCG on STZ-induced changes in (**A**) iNOS protein expressionas well as (b) nitrotyrosine, (**C**) NF-κB and (**B**) TNF-α contents. Each value represents the mean of 6–8 experiments ± S.E.M. *P < 0.05 vs. normal, ^@^*P* < 0.05 vs. EGCG, ^#^*P* < 0.05 vs. STZ.

EGCG administered to normal mice did not affect NF-κB and TNF-α as well as iNOS protein expression compared to the normal control group. Induction of diabetes increased the myocardial NF-κB and TNF-α contents as well as iNOS protein expression, while administration of EGCG preparation to diabetic animals showed marked increment in the previously mentioned markers indicating a severe inflammatory status (Fig. 4).

### EGCG induced apoptosis in diabetic mice

**Figure 5:**
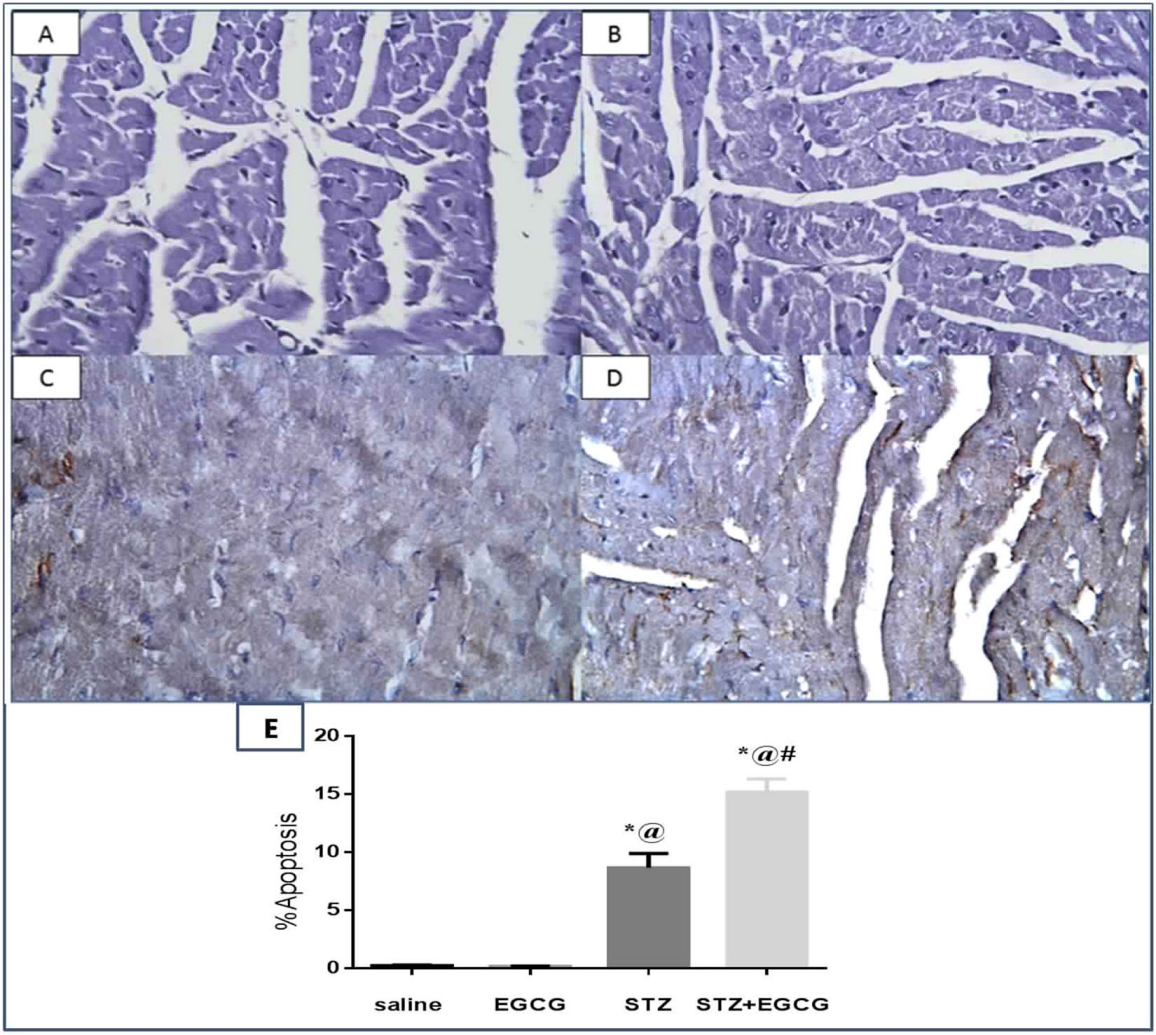
Effect of EGCG on STZ-induced changes in immunohistochemical expression of active caspase-3 expression (400). (**A**) Normal animals receiving saline showed normal myocardium with −ve immunostaining for active caspase-3, (**B**) normal animals receiving EGCG showed normal myocardium with −ve immunostaining for active caspase-3, (**C**) diabetic animals showed focal brown immunostaining for active caspase-3 in myocardial tissues and (**D**) diabetic animals receiving EGCG showed widely distributed brown immunostaining for active caspase-3 in myocardial tissues. Each value represents the mean of 3 experiments ± S.E.M. *P < 0.05 vs. normal, ^@^*P* < 0.05 vs. EGCG, ^#^*P* < 0.05 vs. STZ.

Normal mice treated with EGCG preparation showed no alteration from the normal control animals regarding active caspase-3 expression, whereas diabetic mice receiving EGCG preparation showed significant elevation of active caspase-3 expression compared to diabetic control mice. This signifies EGCG prominent apoptotic potential in the presence of diabetes (Fig. 5).

## Discussion

In an earlier study, we documented the involvement of inflammation, oxidative stress and apoptosis in EGCG-induced nephrotoxicity in the presence of diabetes^19^. To the best of the authors’ knowledge, the current study is the first to provide evidence for the potential cardiotoxic effect of EGCG preparation in diabetic mice. Mouse maybe a better predictor of EGCG toxicity than rat due to higher bioavailability in both human and mouse compared to rat^25,26^. Moreover, i.p route of administration offers a higher bioavailability compared to oral route (poor bioavailability and extensive biotransformation) which allows better investigation of the potential biological activity and toxicity of EGCG as estimated using previous studies^27,28^. Moreover, higher EGCG bioavailability has been linked to higher unconjugated EGCG level which is linked to its related toxicity^29^.

Myocardial hemodynamic and biochemical alterations were clearly observable in the EGCG treated and non treated diabetic groups, which were lower with diabetes alone. Moreover, the microscopic appearances, featuring severe myocardial blood vessels congestion and moderate intermuscular edema in diabetic mice receiving EGCG outweighed the effect of diabetes alone.

The increase in heart to body weight ratio in EGCG treated and non treated diabetic mice indicated hypertrophy of myocardial tissues. Hypertrophy may contribute to impairment of electromotive forces^30^ as demonstrated by decrease in HR and increase in QTc in the present study. HR variability could be also related to metabolic alterations due to hyperglycemia and insulinopenia or downregulation of cardiac adrenergic receptors^31^. Short-term diabetes mellitus induced by STZ has previously demonstrated to modify autonomic control of HR and cause bradycardia which was reversible by insulin administration confirming that these cardiovascular changes were related to metabolic perturbations and not to the direct toxic effect of diabetogenic agent; STZ^32^.

Serum CK-MB is a useful early diagnostic index for myocardial injury and infarction, while troponin-I is a specific and more sensitive cardiac injury biomarker that predicts the risk of both cardiac cell death and subsequent infarction^33^. Therefore, probable reason for the rise in serum levels of CK-MB and troponin-I by EGCG could be cell death, namely apoptosis as will be elaborated later that may impose diabetic mice to the possible risk for myocardial injury. Noteworthy, CK-MB and troponin-I elevations are linked to increased cell permeability caused by inflammation and free radical damage^34,35^. Such effects can be clarified by the core findings of the present work as EGCG preparation, in diabetic mice, activated NFκ-B with the association of TNF-α and NADPH oxidase elevation as well as iNOS overexpression that surpassed diabetic animals. Of note, NADPH oxidase is a major source of ROS^36^ which cause oxidative damage, justified in the present study by the marked increase in oxidative stress markers (TBARS and nitrotyrosine) in addition to marked depletion of endogenous anti-oxidants namely, GSH, GPx, GRx and TAC. The aforementioned events support EGCG deleterious effects in diabetic kidney^19^ and reinforce its reported pro-oxidant effect which was previously linked to its nephro- and hepatotoxicity^16,37^. The elevation of inflammatory cytokines enhances the expression of iNOS contributing to the production of the highly reactive oxidant peroxynitrite in the presence of oxidative stress and resulting in reduced nitric oxide (NO) level and endothelial dysfunction^38^. Of note, the pro-inflammatory cytokine TNF-α^35^ and free radicals^39^ were documented to increase the transcriptional activity of NFκ-B, thus contributing to an endless feed forward cycle that propagates and potentiates both inflammation and oxidative stress leading hence to excessive CK-MB and troponin-I leakage as documented herein in mice receiving EGCG preparation and STZ.

Apart from the involvement of NF-κB in oxidative and inflammatory damage, the current more conspicuous reduced level of HO-1 in EGCG-treated diabetic animals plays also a role in the catechin-mediated myocardial toxicity. This enzyme catalyzes the degradation of heme which has both pro-oxidant and pro-inflammatory properties; hence in the vicinity of a sharp decrease of HO-1, free radicals and inflammation that lead to cellular injury are augmented^40^. The decrease in HO-1 might be clarified by the present inhibition of Nrf2 in EGCG-treated diabetic mice that was again more prominent than diabetic mice. These effects go in line with our previous report of its nephrotoxic effect in the presence of diabetes^19^. Moreover, it has been earlier reported that high doses of EGCG reduced the expression of HO-1^20,41^. The transcription factor Nrf2 is an important machinery that upregulates the HO-1 gene. This enzyme initiates antioxidant and anti-inflammatory processes, hence Nrf2 down-regulation triggers and progresses various diabetic complications^42,43^. Moreover,it was reported that STZ-induced diabetes in Nrf2 knockout mice rapidly progressed to severe myocardial lesions associated by robust inflammatory response, oxidative stress, and apoptosis^44^. These data indicate that Nrf2 and its downstream target HO-1 serve as defense factors against cardiac diabetic complications; hence their excessive reduction by EGCG preparation in diabetic animals seen in this study can explain, in part, the polyphenol cardiotoxic effect. Besides the upregulation of HO-1, Nrf2 initiates the transcription of a number of anti-oxidative genes to reduce the pathological oxidative stress in the heart^43^. Such an effect might afford an additional explanation to the previously documented descent of TAC and GSH in addition to antioxidants enzymes controlling GSH levels (GPx and GR) by EGCG + STZ in the present work.

In response to various forms of stress, cells activate a highly conserved heat shock response in which a set of HSPs are induced to participate in cellular repair and protective mechanisms^45,46^. Several studies noticed that diabetes is associated with significant reduction of HSPs levels^47–49^. Moreover, in diabetic patients, cells are more vulnerable to damage if HSPs levels are decreased which results in organ failure^50^. Since in the present study EGCG preparation was shown to suppress HSP 90 in diabetic mice more persistently than diabetes alone, therefore, EGCG-induced cardiotoxicity could be attributed to its ability to decrease HSP 90, and hence maladapted stress defense responses. The present decrease in HSP 90 after EGCG administration in diabetic mice confirms our previous report in the kidney^19^ and is consistent with the previous finding which elucidated that this proteo-stress marker was markedly down-regulated in mice receiving high doses of green tea polyphenols in diet^16^. Furthermore, in response to excessive oxidative stress-induced by high doses of EGCG, HSP90 was downregulated in liver and kidney^20^.

Diabetic control animals have shown low incidence of apoptosis; however, active caspase-3 was prominently elevated in the STZ/EGCG group, solidifying its apoptotic potential in the diabetic kidney^19^. It has been anticipated that oxidative stress and the pro-inflammatory cytokine TNF-α could contribute to diabetic cardiomyopathy through the stimulation of the intrinsic and extrinsic pathways of programmed cell death, respectively^51^. Additionally, Kim *et al*.^52^. suggested that the decreased HSP 90 expression in type 2 diabetic kidneys may mediate apoptosis; hence affording an additional clarification for increased apoptosis associated by EGCG administration in STZ mice. Accordingly, in the present study, the excessive cardiac toxicity produced by the polyphenol under hyperglycemic conditions could been linked to the present exaggerated oxidative stress and inflammation responses, as well as the reduction of HSP 90 in the heart to jeopardize cardiomyocytes function leading to CK-MB and troponin-I elevation as previously anticipated.

Therefore, the current investigation elaborated that parentral application of EGCG preparation (Teavigo^®^) exhibited cardiotoxicity in the presence of diabetes although no toxicity was observed in normal animals receiving the same dose and route of administration. This deleterious effect could be attributed to EGCG-induced apoptosis triggered by oxidative stress, inflammation and HSP 90 suppression which eventually leads to cardiac damage evidenced by elevation of myocardial biomarkers.

## Conclusion

In spite of its well-known favorable effects, EGCG administration to diabetic mice revealed increased cardiotoxicity. Considering the rise of global diabetes, EGCG administration may impact cardiovascular health accelerating the progression of the disease-associated complications. Mediation of oxidative stress, inflammation, and apoptosis in the cardiomyopathy-induced by EGCG preparation in diabetes can be considered especially for parentral application of EGCG which exhibits higher bioavailability and thus its related toxicity. Accordingly, diabetic patients should consume EGCG based supplements cautiously until their cardiotoxic effects in the presence of diabetes are clinically evaluated.

### Limitations and recommendations for future research

Despite being 94% of teavigo content, further studies are required to investigate the toxicity of pure EGCG in presence of uncontrolled hyperglycemic conditions and complications of chronic untreated diabetes.

## Electronic supplementary material

Supplementary Information
