# Supplementary material for: Paradoxical cardiotoxicity of intraperitoneally-injected epigallocatechin gallate preparation in diabetic mice
Source: Sci Rep. 2018 May 18;8:7880. doi: 10.1038/s41598-018-25901-y (PMC5959847; doi:10.1038/s41598-018-25901-y)
Supplement: Supplementary file 1 — Supplementary Information [file 41598_2018_25901_MOESM1_ESM.docx]

***Paradoxical cardiotoxicity of intraperitoneally-injected epigallocatechin gallate preparation in diabetic mice***

## Authors

***Nora O. Abdel Rasheed^1^, LamiaaA. Ahmed^2^, Dalaal M. Abdallah^3^,** [**Bahia M. El-Sayeh**](http://www.pubfacts.com/author/Bahia+M+El-Sayeh)**^4^**

**Corresponding author: Nora O. Abdel Rasheed**^1^

**Email of corresponding author**:**nora17889@yahoo.com**

1. Department of Pharmacology and Toxicology, Faculty of Pharmacy, Cairo University, Egypt.
2. Department of Pharmacology and Toxicology, Faculty of Pharmacy, Cairo University, Egypt.
3. Department of Pharmacology and Toxicology, Faculty of Pharmacy, Cairo University, Egypt.
4. Department of Pharmacology and Toxicology, Faculty of Pharmacy, Cairo University, Egypt.

**Supplementary table 1: Effect of different doses of EGCG (50,100,150 and200 mg/kg) on percentage of mortality and serum troponin-I.**

|  | **Normal control (saline)** | **EGCG 50** | **EGCG 100** | **EGCG 150** | **EGCG 200** |
| --- | --- | --- | --- | --- | --- |
| **Mortality %** | 0% | 0% | 0% | 40% | 100% |
| **Serum troponin-I (ng/ml)** | 0.65 ± 0.03 | 0.43 ± 0.01* | 0.59 ± 0.02 | 1.56 ± 0.04* | Not estimated as all animals died |

Each value represents the mean of 5-8 experiments ± S.E.M.

* Significantly different from normal group at *p <0.05*
